# R-spondin-2 is a Wnt agonist that regulates osteoblast activity and bone mass

**DOI:** 10.1038/s41413-018-0026-7

**Published:** 2018-08-14

**Authors:** M. Noelle Knight, Kannan Karuppaiah, Michele Lowe, Sarthak Mohanty, Robert L. Zondervan, Sheila Bell, Jaimo Ahn, Kurt D. Hankenson

**Affiliations:** 1 Department of Orthopaedic Surgery, Perelman School of Medicine, University of Pennsylvania, Philadelphia, USA; 2 Department of Orthopaedic Surgery, University of Michigan Medical School, Ann Arbor, USA; 3 Division of Pulmonary Biology, Cincinnati Children’s Hospital Medical Center, Cincinnati, USA

## Abstract

The R-spondin family of proteins are Wnt agonists, and the complete embryonic disruption of *Rspo2* results in skeletal developmental defects that recapitulate the phenotype observed with *Lrp5/6* deficiency. Previous work has shown that R-spondin-2 (*Rspo2*, RSPO2) is both highly expressed in Wnt-stimulated pre-osteoblasts and its overexpression induces osteoblast differentiation in the same cells, supporting its putative role as a positive autocrine regulator of osteoblastogenesis. However, the role of Rspo2 in regulating osteoblastogenesis and bone formation in postnatal bone has not been explored. Here we show that limb-bud progenitor cells from *Rspo2* knockout mice undergo reduced mineralization during osteoblastogenesis in vitro and have a corresponding alteration in their osteogenic gene expression profile. We also generated the first *Rspo2* conditional knockout (Rspo2^floxed^) mouse and disrupted *Rspo2* expression in osteoblast-lineage cells by crossing to the Osteocalcin-Cre mouse line (Ocn-Cre + Rspo2^f/f^). Ocn-Cre + Rspo2^f/f^ male and female mice at 1, 3, and 6 months were examined. Ocn-Cre + Rspo2^f/f^ mice are decreased in overall body size compared to their control littermates and have decreased bone mass. Histomorphometric analysis of 1-month-old mice revealed a similar number of osteoblasts and mineralizing surface per bone surface with a simultaneous decrease in mineral apposition and bone formation rates. Consistent with this observation, serum osteocalcin in 3-month-old Ocn-Cre + Rspo2^f/f^ was reduced, and bone marrow-mesenchymal stem cells from Ocn-Cre + Rspo2^f/f^ mice undergo less mineralization in vitro. Finally, gene expression analysis and immunohistochemistry of mature bone shows reduced beta-catenin signaling in Ocn-Cre + Rspo2^f/f^. Overall, RSPO2 reduces osteoblastogenesis and mineralization, leading to reduced bone mass.

## Bone Physiology: R-spondin-2 reduces mineralization, leading to decreased bone mass

A loss of R-spondin-2 reduces osteoblastogenesis (production of osteoblasts, the cells from which bone develops) and mineralization, thereby leading to decreased bone mass in adults. R-spondin-2 is one of a family of four proteins that are expressed in the developing mouse limb as well as other tissues; each R-spondin family member exerts a different functional effect. R-spondins clearly influence several aspects of skeletal biology, but their specific roles—especially in postnatal bone—remained to be elucidated. A team headed by Kurt Hankenson at the University of Michigan Medical School investigated the role of R-spondin-2 in osteoblastogenesis, both in vitro and in vivo, using a mouse model. For the first time, the team was able to demonstrate that R-spondin-2 promotes osteoblastogenesis, bone development, and consequent bone mass growth in adult mice.

## Introduction

Bone remodeling is critical for the maintenance of bone mass and structure, and is particularly important when considering pathologies that disrupt homeostasis, such as those that lead to the development of low-bone mass and osteoporosis. This decrement in bone mass and structure, and the concordant decrease in mechanical strength can be caused both by dysregulation of osteoblastic and osteoclastic activity.^1^ Therapeutics to restore bone mass and strength therefore focus on targeting one of these two processes.

A potent positive regulator of bone deposition by osteoblasts during bone modeling and remodeling is the canonical Wnt signaling pathway, making it a highly desirable target for therapeutics to increase bone formation. The Wnt pathway has been deeply investigated in the context of bone, focusing on both extracellular pathway modulators and the stabilization of beta-catenin and transcription factors, generally with increases in canonical signaling through the pathway leading to increased osteoblastogenesis and bone formation.^2^ Modulation of the pathway through antibodies targeting Sclerostin, an antagonist of canonical Wnt signaling in bone, has been particularly successful.^3^ These antibodies, along with PTH and PTHrP analogs teriparatide and abaloparatide, are currently the only FDA-approved osteoanabolic therapeutics; other interventions primarily focus on inhibiting further resorption of bone by osteoclasts, such as the bisphosphonates.^4^ There remains a critical need for the identification of additional osteoanabolic therapeutic options given the clinical limitations of the currently available therapeutics.

The R-spondin proteins, a family of four secreted matricellular proteins, potentiate Wnt signaling and may represent a novel focus of therapeutic targeting to modulate osteoblasts. The R-spondins activate canonical Wnt signaling by binding to the Leucine-Rich Repeat-Containing G-Protein Coupled Receptors 4–6 (LGR4-6), which then interacts with the E3-Ubiquitin ligases ZNRF3/RNF43 to inhibit Wnt receptor turnover.^5,6^ R-spondins are also reported to activate non-canonical Wnt/Planar Cell Polarity (PCP) signaling.^7^ All four R-spondin family members have a similar modular domain structure: an N-terminal signal sequence, two furin-like/cysteine-rich domains, a thrombospondin type-1 motif (TSR1), and a basic amino acid rich C-terminus, which are encoded on separate exons.^8^ Each of the R-spondins are expressed in the developing mouse limb, as well as in other tissues, and each has different functional effects with respect to the skeleton. R-spondin-1 (*Rspo1*) disruption is not associated with a skeletal phenotype, but its expression is induced during osteoblastogenesis and in response to vibration.^9^ The *Rspo2* knockout mouse (Rspo2^ftl^) is perinatal lethal due to defective lung development and exhibits limb and craniofacial malformations.^10,11^ Our laboratory has previously demonstrated that *Rspo2* is highly expressed in Wnt11-overexpressing pre-osteoblasts. Furthermore, overexpression of *Rspo2* enhanced osteoblastogenesis in vitro.^12^ *Rspo3* disruption is embryonic lethal due to angiogenic defects in the murine placenta, though conditional knockout in limb mesenchyme (via Prx1-Cre recombinase-mediated disruption) exacerbated *Rspo2* limb malformations in double knockouts.^13–15^ *Rspo4* mutations in humans lead to anonychia, but a skeletal phenotype has not been identified.^16,17^ It is clear that R-spondins influence several aspects of skeletal biology, but their cell-specific roles, particularly in post-natal bone have yet to be elucidated.

Herein, the clear anabolic in vitro effects of *Rspo2* on osteoblastogenesis led us to further investigate the role of *Rspo2* in osteoblastogenesis. We utilized limb bud progenitor cells from the global knockout Rspo2^ftl^ (*Footless*) mouse model to evaluate *Rspo2*-deficient osteoblastogenesis in vitro. Further, due to the perinatal lethality of this model, we generated a conditional floxed *Rspo2* allele. We crossed this model to a pan-expressing Cre mouse (EIIa-cre) and recapitulated the complete knockout phenotype. Next we crossed the floxed mouse to a mouse line that expresses Cre-recombinase under the control of the Osteocalcin (Ocn) promoter for specific recombination in osteoblasts to determine the function of *Rspo2* in osteoblasts in postnatal bone. At 1 month, 3 months, and 6 months of age, we evaluated the skeletal phenotype of these mice by micro-coputered tomography(μCT), histomorphometry, mechanical testing, and analysis of progenitor cell populations. For the first time, we show that RSPO2, a Wnt signaling agonist, impacts osteoblastogenesis, bone development and adult bone mass accrual.

## Results

### Rspo2-null limb-bud mesenchymal progenitor cells from Footless mice have defective osteoblastogenesis

**Fig. 1:**
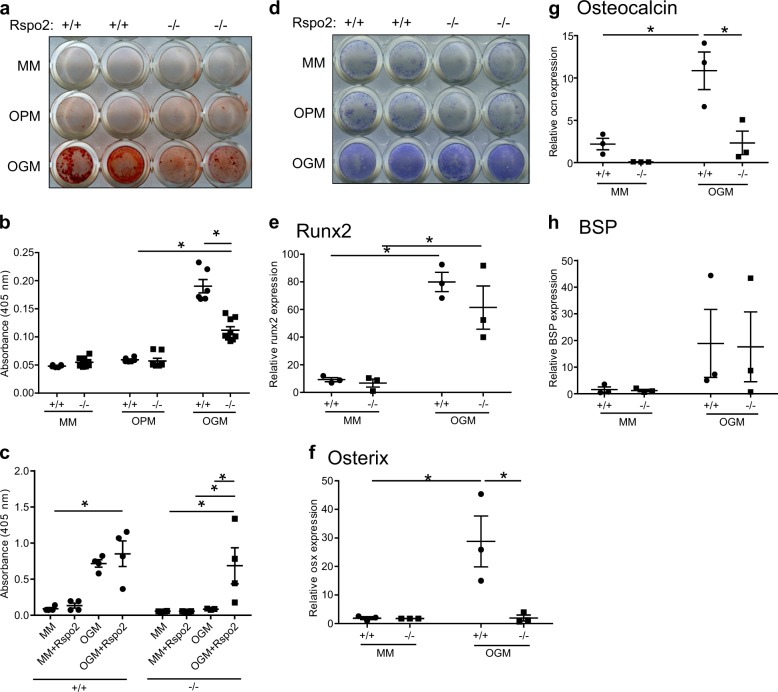
R-spondin-2 null mesenchymal progenitor cells from *Footless* mutant mice show reduced osteoblastogenesis. **a** Alizarin red S staining of representative wells from in vitro osteogenesis assay after 10 days. **b** Overall quantification of Alizarin Red S staining of osteogenesis at 10 days, as shown in **a**. **c** Quantification of osteogenesis at 15 days with addition of recombinant hRSPO2 (10 nmol·L^-1^). **d** Alkaline Phosphatase (ALP) staining of similar osteogenesis assay at 5 days. **e** Relative Runx2 expression after 5 days of osteogenesis in vitro. **f** Relative Osterix expression after 5 days of osteogenesis in vitro. **g** Relative Osteocalcin expression after 10 days of osteogenesis in vitro. **h** Relative bone sialoprotein (BSP) expression after 5 days of osteogenesis in vitro. (*n* = 3 cell lines from individual animals, assay performed in triplicate for each cell line (sex of pre-natal pups not determined), * indicates  *P*< 0.05. MM maintenance media, OPM osteopermissive media with β-glycerophosphate and ascorbic acid-2-phosphate, OGM osteogenic media with OPM and 2.5 nmol·L^-1^ rhBMP-6

Our previous work demonstrated that *Rspo2* was required for Wnt11-mediated osteoblastogenesis in MC3T3E1 cells, but the autocrine role of RSPO2 in primary osteoblasts was not examined.^12^ MSC were harvested from Rspo2^Ftl^ mouse limb bud mesenchyme. These cells undergo osteoblast differentiation in the presence of BMP but not with ascorbic acid and inorganic phosphate alone. MSC isolated from Rspo2^Ftl^ mice (deficient in RSPO2) showed reduced osteoblastogenesis (Fig. 1). Mineralization was significantly reduced in Rspo2^Ftl^ MSC compared to MSC from wild-type littermates (Fig. 1a, b), a difference that could be rescued with the addition of recombinant RSPO2 to the media (Fig. 1c). Despite the contrast in mineralization, there was no difference in alkaline phosphatase staining between the genotypes (Fig. 1d).

To better understand the mechanism of reduced mineralization in the *Rspo2*-deficient cells, osteogenic gene expression was studied using RT-qPCR. There was no difference in the expression of *Runx2*, the master regulator of osteogenesis (Fig. 1e). However, the downstream osteoblast-associated transcription factor, Osterix (*Osx*), was significantly reduced in the Rspo2^Ftl^ MSC after 5 days of osteogenesis (Fig. 1f) and remained decreased over the course of osteoblastogenesis (data not shown). The late-stage, osteoblast-secreted protein, Osteocalcin (*Ocn*), had reduced gene expression in the Rspo2^Ftl^ cells at 10 days of differentiation (Fig. 1g), though this difference diminished with further differentiation (data not shown). In contrast, bone sialoprotein (*Bsp*), another late-stage osteoblastogenesis gene that is involved in the mineralization of the matrix, did not show differences in expression in the Rspo2^Ftl^ MSC compared to the wild-type control cells (Fig. 1h).

### Generation of R-Spondin-2 Conditional Allele

**Fig. 2:**
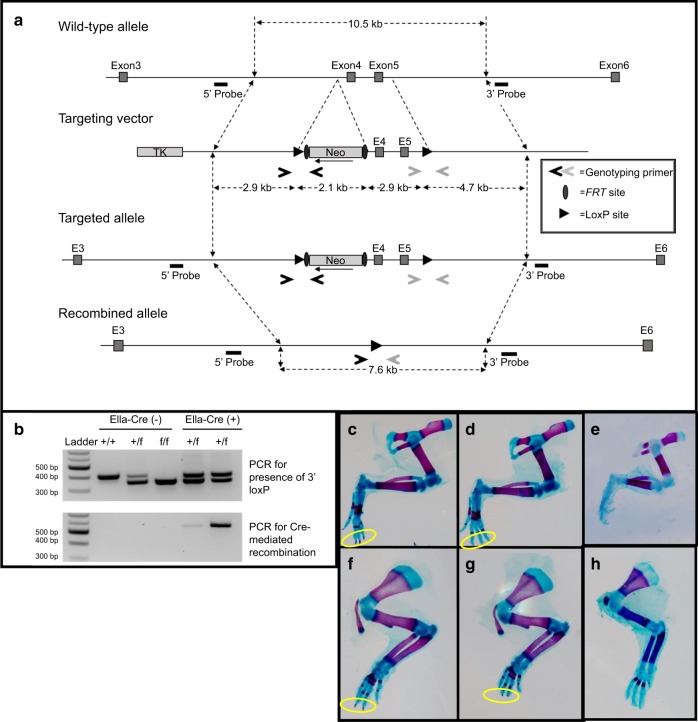
Generation of R-spondin-2 Conditional Allele. **a** Map of the genomic region of exons 3–6 of *Rspo2* in alignment with the targeting vector with exons 4 and 5 flanked by loxP sites (triangles), as well as the addition of a neomycin cassette flanked by FRT sites. The targeted allele after homologous recombination follows, which is then in alignment with the final recombined allele after Cre recombination of the loxP sites. Genotyping primers are shown as open arrows (Primer set **a** dark-grey; Primer set **b**, light-grey), and Southern blot probes are denoted. **b** Genotyping of mice with primers Rspo2-FloxB-for, Rspo2-FloxB-rev, and Rspo2-FloxA-For. Pelvic limbs (**c**–**e**) and thoracic limbs (**f**–**h**) from embryonic skeletal preparations. Limbs from representative WT embryos shown in **c**, **f**; **d**, **g**, from most minorly affected EIIa-Cre+ Rspo2^f/f^embryos; **e**, **h** from more severely affected embryos. **c**, **d** and **f**–**h** from E18.5 embryos, **e** from E16.5 embryo. Yellow circles highlight the digital tips. Note the absence of red staining at the tips showing delayed mineralization. Ella-Cre-Rspo2^f/+^, *n* = 12; EIIa-Cre + Rspo2^f/+^, *n* = 13; Ella-Cre-Rspo2^f/f^, *n* = 10; EIIa-Cre + Rspo2^f/f^, *n* = 15 (sex of pre-natal pups was not determined)

The perinatal lethality of the *Rspo2* knockout mice prevents the examination of the impact of *Rspo2* on osteoblastogenesis and bone formation in post-natal bone. The R-spondin-2 allele proved difficult to target using the most common strategies for loxP site selection as the gene contains very large introns that are in-frame. Thus, we targeted the disruption of the Furin and TSR repeats of the R-spondin-2 gene (Fig. 2a). Previous work has shown that such disruption results in a complete suppression of beta-catenin-mediated function.^18^ The floxed allele yields a 359 bp fragment, while the wild-type allele yields a 413 bp fragment. The genotyping can also be combined with the forward primer from the upstream loxP site for determination of recombination when Cre recombinase is present, which yields a 512 bp fragment (Fig. 2b).

### Recapitulation of Rspo2^ftl^ phenotype with Ella-Cre expression

To test the functional significance of the *Rspo2* allele in vivo, Rspo2^floxed^ mice were crossed with the pan-Cre expressing EIIa-cre mouse line, which has whole-body Cre-recombinase expression when the allele is inherited from the dam. Three litters from f/f Cre-negative X f/wt Cre-positive matings were aged to weaning. At P21, no EIIa-Cre + Rspo2^f/f^ mice were identified. The genetic frequency was consistent with perinatal lethality of the EIIa-Cre + Rspo2^f/f^ mice, similar to the Rspo2^Ftl^ global knockout (Tables 1–2). The other possible genotypes each accounted for approximately one-third of the total offspring. Skeletal preparations of E16.5 and E18.5 pups from a similar mating scheme were stained with Alcian blue and Alizarin red S. All Ella-Cre + Rspo2^f/f^ pups had dysmorphic distal phalanges with decreased mineralization (10/10 embryos), and several pups additionally had reduced (8/10 embryos) or absent digits (4/10 embryos) (Fig. 2c–h). This is consistent with the phenotype of the three models of *Rspo2* global knockouts that have been previously published.^10,11,19–21^ As may be expected because of inconsistency in Cre-mediated knockout relative to the complete knockout, there was heterogeneity in the embryo phenotypes. Figure 2d and g shows the least severe phenotypes of the pelvic and thoracic limbs, respectively from targeted mice, with reduced distal phalangeal mineralization in the distal limb. Figure 2e and h are representative images showing limbs of more severely affected individuals. Figure 2e is a E16.5 embryo with pronounced foot dysmorphism of the pelvic limb, while Fig. 2h is a thoracic limb lacking a clavicle and with digital dysmorphism.

**Table 1:** Genetic frequency of P18.5 pups resulting from EIIa-Cre-Rspo2^f/f^ x EIIa-Cre+Rspo2^f/+^ matings

Items	f/+; Cre-	f/+; Cre+	f/f; Cre-	f/f; Cre+
Frequency	0.25	0.17	0.17	0.42
*N*	6	4	4	10

**Table 2:** Genetic frequency of P21 pups resulting from EIIa-Cre-Rspo2^f/f^ x EIIa-Cre+Rspo2^f/+^ matings

Items	f/+; Cre-	f/+; Cre+	f/f; Cre-	f/f; Cre+
Frequency	0.36	0.29	0.36	0
*N*	5	4	5	0

### Osteoblast-specific knockout results in decreased size and decreased bone mass

**Fig. 3:**
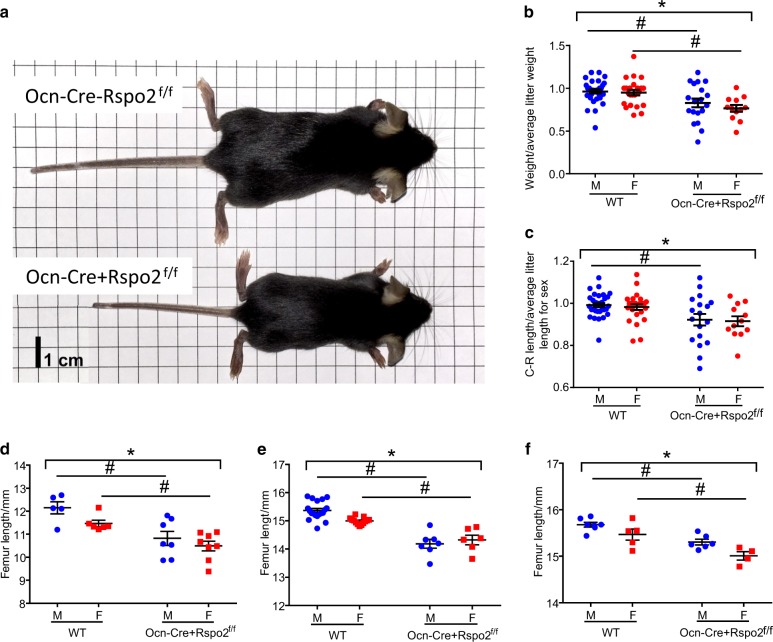
Osteoblast-specific *Rspo2* knockout mice have decreased body size. **a** Comparison of male WT (top) vs. Ocn-Cre + Rspo2^f/f^ (bottom) mice at 3 weeks (weaning). **b** Normalized weights of 3-week-old mice. WT males, *n* = 31; Ocn-Cre + Rspo2^f/f^ males, *n* = 19; WT females, *n* = 24; Ocn-Cre + Rspo2^f/f^ females, *n* = 12. **c** Normalized Crown-Rump length of 3 weeks old mice. WT males, *n* = 31; Ocn-Cre + Rspo2^f/f^ males, *n* = 19; WT females, *n* = 25; Ocn-Cre + Rspo2^f/f^ females, *n* = 12. **d** Femur length of 1-month-old mice. WT males, *n* = 5; Ocn-Cre + Rspo2^f/f^ males, *n* = 7; WT females, *n* = 6; Ocn-Cre + Rspo2^f/f^ females, *n* = 8. **e** Femur length of 3-month-old mice. WT males, *n* = 19; Ocn-Cre + Rspo2^f/f^ males, *n* = 7; WT females, *n* = 13; Ocn-Cre + Rspo2^f/f^ females, *n* = 6. **f** Femur length of 6-month-old mice. WT males, *n* = 7; Ocn-Cre + Rspo2^f/f^ males, *n* = 6; WT females, *n* = 5; Ocn-Cre + Rspo2^f/f^ females, *n* = 4. *indicates  *P*< 0.05 for genotype groups. # indicates  *P*< 0.05 for genotypes within sex. Blue circles = males; Red circles (**b**) or squares (**c**–**f**)  = females

As our research has previously shown, Rspo2 is highly expressed by osteoblasts in vitro.^12^ In adult bone Rspo2 is expressed highly by bone lining cells, osteoblasts, and osteocytes, but is not expressed in growth plate chondrocytes (Supplementary Figure S1). To ask about the osteoblast-specific role of Rspo2, Rspo2^floxed^ mice were crossed with the well-characterized Osteocalcin-Cre mice to disrupt *Rspo2* selectively in mature osteoblasts.^22^ Mice were measured (length and weight) at 3 weeks of age and then harvested at 1 month, 3-month, and 6-months of age for skeletal analysis of juvenile, young adult, and mature adult bone, respectively. At 3 weeks of age, targeted (homozygous Rspo2^floxed^, Ocn-Cre-positive, herein referred to as Ocn-Cre + Rspo2^f/f^) mice weighed less and had shorter crown-rump measurements than their control (homozygous Rspo2^floxed^, Ocn-Cre-negative, herein referred to as WT) littermates (Fig. 3a–c). At all timepoints, the femurs of Ocn-Cre + Rspo2^f/f^ mice were significantly shorter than those of their control littermates (Fig. 3d–f), but relative change in limb length from 1-month to 3-months and 3-months to 6-months was unchanged between WT and Ocn-Cre + Rspo2^f/f^.

**Fig. 4:**
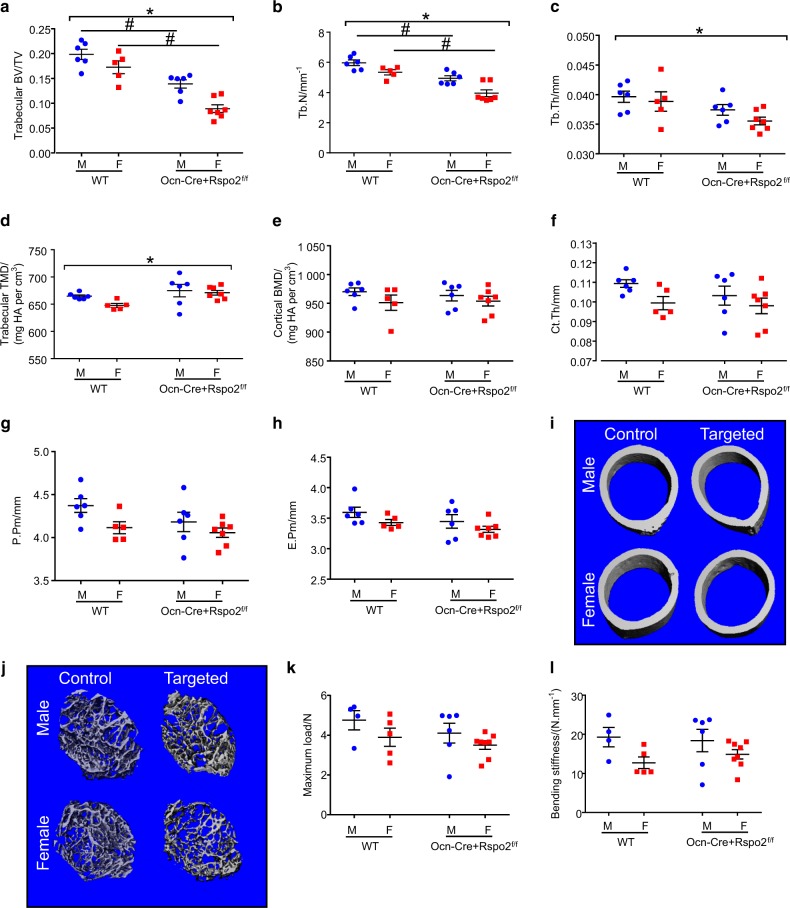
Targeted mice have decreased trabecular and cortical bone parameters. **a**–**h** Micro-computed tomography (uCT) of femurs from 1-month-old mice. Femurs were analyzed for bone volume fraction (**a**), trabecular number (**b**), trabecular thickness (**c**), trabecular bone mineral density (**d**), cortical bone mineral density (**e**), cortical thickness (**f**), periosteal perimeter (**g**), and endosteal perimeter (**h**). WT males, *n* = 6; Ocn-Cre + Rspo2^f/f^ males, *n* = 6; WT females, *n* = 5; Ocn-Cre + Rspo2^f/f^ females, *n* = 7. **i**, **j** Three dimensional reconstructions of mid-diaphyseal cortical bone (**i**) and metaphyseal trabecular bone (**j**) from representative 1-month old mice. 3-point bending of femurs from 1-month-old mice. Femurs were analyzed for (**k**) Maximum Load and (**l**) Bending Stiffness. WT males, *n* = 4; Ocn-Cre + Rspo2^f/f^ males, *n* = 6; WT females, *n* = 5; Ocn-Cre + Rspo2^f/f^ females, *n* = 8. *indicates  *P*< 0.05 for genotype groups. # indicates  *P*< 0.05 between genotypes within sex

Microcomputed tomography (μCT or microCT) was used to evaluate trabecular and cortical bone of the right femur from mice at all timepoints for both sexes (Fig. 4 and Figures S2 and S3). At 1 month of age, Ocn-Cre + Rspo2^f/f^ mice had decreased trabecular BV/TV compared to their control littermates (Fig. 4a). The decreased BV/TV ratios corresponded to both decreased trabecular number and decreased trabecular thickness (Fig. 4b–c, j). The tissue mineral density of the trabecular bone and cortical bone did not differ across genotypes (Fig. 4d, e). At 1 month of age, no significant differences were identified in the cortical bone parameters (Fig. 4f–i).

At 3 months the phenotype had become more pronounced. Trabecular bone volume fraction (BV/TV) was significantly decreased in the Ocn-Cre + Rspo2^f/f^ mice, with trabecular thickness being the main contributor to this difference (Supplementary Figures S2A-C, J). The difference in trabecular bone was more pronounced in male mice, with female targeted mice only trending to have decreased trabecular bone. Trabecular and cortical tissue mineral densities were not different between groups (Supplementary Figures S2D,E). In cortical bone, 3-month-old mice had decreased cortical bone volume, decreased cortical thickness, decreased endosteal and periosteal perimeters, and decreased polar moment of inertia (Supplementary Figures S2F-I). However, when males and females are considered separately, only targeted male mice showed significant reductions in these parameters.

At 6 months of age, Ocn-Cre + Rspo2^f/f^ mice demonstrated decreased trabecular BV/TV, which was again primarily due to decreased trabecular thickness rather than trabecular number (Supplementary Figures S3A-C, J). The tissue mineral densities of trabecular and cortical bone were not different between groups (Supplementary Figures S2D-E). While cortical BV and cortical thickness still showed a decreasing trend in the Ocn-Cre + Rspo2^f/f^ mice at 6 months, these parameters were no longer statistically significant (Supplementary Figures S3F,I). Endosteal and periosteal perimeters and polar moment of inertia were not different between groups (Supplementary Figure S3G-H, data not shown).

Representative three-dimensional reconstructions of trabecular and cortical bone of mice from individual mice that had BV/TV and cortical thickness most similar to the median group values are shown (Fig. 4i, j, Supplementary Figures S2I-J, S3I-J). While statistically significant differences in bone phenotype exist between male and female mice of both control and Rspo2^f/f^ mice, they are consistent with well-established sex differences in bone geometry in mice and are not reported herein.

Right femora were subjected to biomechanical testing via 3-point bending. There were no differences in the maximum load force or bending stiffness at 1 month of age when cortical bone geometry was similar (Fig. 4k, l). At 3 months of age and 6 months of age Ocn-Cre + Rspo2f/f mice had decreased maximum load force and decreased bending stiffness (Supplemental Figures 2k-l and 3k-l).

### Rspo2 knockout mice have decreased bone formation

**Fig. 5:**
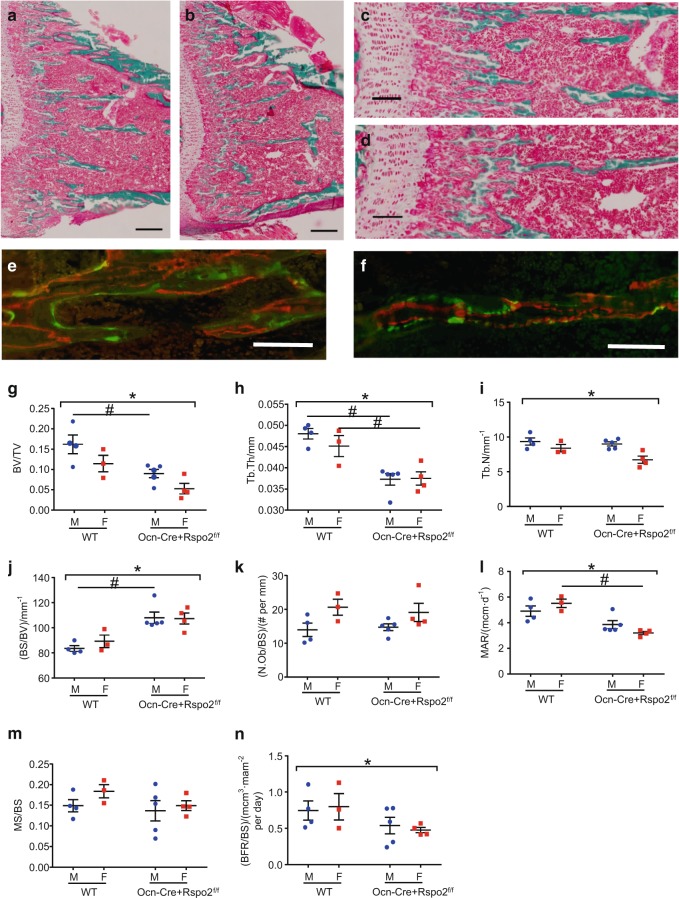
Ocn-cre knockout of *Rspo2* results in decreased bone formation. **a**–**d** Histologic sections of the proximal tibia of representative 1-month-old mice stained with Goldner’s Trichrome. **a**, **c** WT control mice. **b**, **d** Ocn-Cre + Rspo2^f/f^ mice. **e**, **f** Double-labeled bone sections from representative 1-month-old mice. **e** WT control mouse. **f** Ocn-Cre + Rspo2^f/f^ mouse. **i**–**l** Histomorphometric parameters from 1-month-old mice. Tibias were analyzed for bone volume fraction (**g**), trabecular thickness (**h**), trabecular number (**i**), bone surface (**j**), osteoblasts per bone surface (**k**), mineral apposition rate (**l**), mineralizing surface (**m**), bone formation rate (**n**). WT males, *n* = 4; Ocn-Cre + Rspo2^f/f^ males, *n* = 5; WT females, *n* = 3; Ocn-Cre + Rspo2^f/f^ females, *n* = 4. Scale bars are 166 μmol·L^-1^ in (**a**–**d**); 100 μmol·L^-1^ in **e**–**h**. *indicates *P* < 0.05 for genotype groups. # indicates *P* < 0.05 for different genotypes within sex. Blue circles = male; red squares = females

Static and dynamic histomorphometric analysis of trabecular bone of proximal tibiae from 1-month-old mice was performed to further assess bone microarchitecture as well as to assess bone formation. Representative histologic sections from WT (Fig. 5a, c) and Ocn-Cre + Rspo2^f/f^ (Fig. 5b, d) littermate mice are shown. Calcein/Alizarin double-labeled bone sections from WT (Fig. 5e) and Ocn-Cre + Rspo2^f/f^ mice (Fig. 5f), respectively, are also shown. Similar to the microCT analysis, targeted mice had decreased trabecular BV/TV (Fig. 5g) primarily due to a corresponding decrease in trabecular thickness (Fig. 5h), though there is a small but significant reduction in the trabecular number as well (Fig. 5i). Consistent with this, the bone surface to bone volume ratio was increased in Ocn-Cre + Rspo2^f/f^ mice (Fig. 5j). However, while the total number of osteoblasts was reduced in the targeted animals (not shown), the number of osteoblasts per bone surface was not different between groups (Fig. 5k). Additionally, we measured the interlabel distance between the two fluorescent bone labels to calculate the mineral apposition rate. The mineral apposition rate was significantly decreased in the Ocn-Cre + Rspo2^f/f^ mice compared to their control littermates (Fig. 5l). The percent mineralizing surface (MS/BS) was calculated by measuring the fluorescently single- and double-labeled surfaces relative to the total surface distance, and this was also not different between the groups (Fig. 5m). However, it should be noted that the overall extent of label was much greater in the WT do to the greater amount of WT bone (single labeled and double labeled surfaces) (not shown). The bone formation rate (BFR/BS) was calculated to assess the combined contributions of mineralizing surface and mineral apposition rate. The BFR was reduced in the Ocn-Cre + Rspo2^f/f^ mice (Fig. 5n).

Similar evaluation of 3-month-old mice by histomorphometry revealed that BV/TV (Supplementary Figure S4A) and trabecular parameters (Supplementary Figure S4B,C) were reduced, consistent with microCT results, but at 3-months MAR and BFR were not significantly different (Supplementary Figure S4 E,G). Similar to the 1-month-old mice, the MS/BS in the Ocn-Cre + Rspo2^f/f^ mice was not changed (Supplementary Figure S4F), despite significant reductions in overall sLS and dLS in the targeted mice (not shown), because the 3-month-old Rspo2-deficient mice have reduced total bone surface. While there were changes in bone formation, there were no apparent changes in TRAP stained osteoclasts evaluated using histomorphometry in 3-month-old Ocn-Cre + Rspo2^f/f^ mice (Supplementary Figure S4H-L).

Serum measures of bone formation and resorption were also assessed (Supplementary Figure S5) Serum osteocalcin, a measure of bone formation, was decreased in 3-month-old Ocn-Cre + Rspo2^f/f^ mice (Supplementary Figure S5A), but TRAP, a measure of bone resorption was unchanged (Supplementary Figure S5C), which is consistent with the osteoclast histomorphometry. On the other hand, at 6-months of age osteocalcin levels were unchanged, with WT levels dropping to the level of Ocn-Cre + Rspo2^f/f^ mice (Supplementary Figure S5B), while serum TRAP levels were increased in the 6-month-old Ocn-Cre + Rspo2^f/f^ mice (Supplementary Figure S5D).

### Decreased progenitor cell population and mineralization of BM-MSC from the conditional knockout mouse

**Fig. 6:**
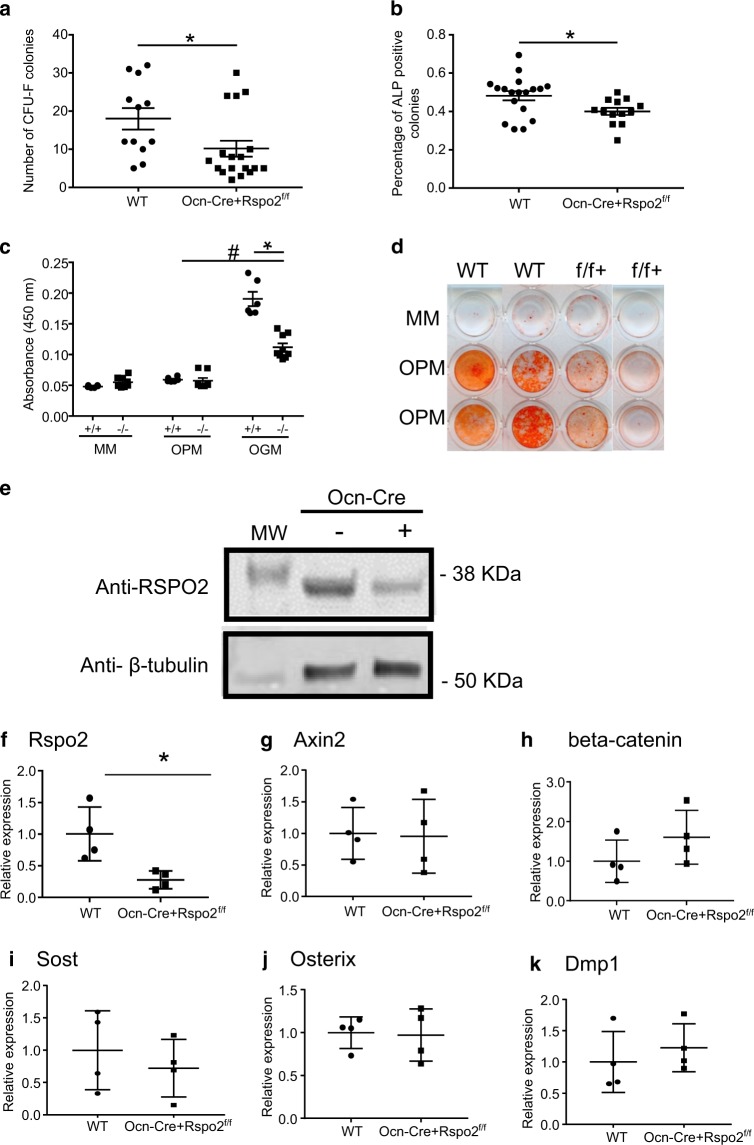
Osteoblast-specific *Rspo2* knockout mice show decreased progenitor cells and BM-MSC mineralization. **a** Quantification of total number of CFU-F colonies from bone marrow flushed from 1-month-old male and female mice. **b** Quantification of percent ALP-positive colonies from bone marrow flushed from 1-month-old male and female mice. WT, *n* = 12 (*n* = 6 male; *n* = 6 females); Ocn-Cre + Rspo2^f/f^, *n* = 18 (*n* = 8 males; *n* = 10 females). **c** Quantification of Alizarin red S staining of 10 day osteogenesis with BM-MSC from 1-month-old male and female mice. **d** Alizarin Red S staining of representative wells after 10 days of osteogenesis. WT, *n* = 8 (*n* = 4 male; *n* = 4 female); Ocn-Cre + Rspo2^f/f^, *n* = 8 (*n* = 4 male; *n* = 4 female). **e** Western blot for Rspo2 protein. **f**–**h** Quantitative gene expression of BM-MSC in culture. *n* = 4 males for each genotype. **f** Rspo2 expression. **g** Axin2 expression. **h** beta-catenin expression. **i** Sost expression. **j** Osterix expression. **k** Dmp1 expression. *indicates *P* < 0.05 for genotype groups. # indicates *P* < 0.05 for OPM vs OGM media. MM maintenance media, OPM osteopermissive media with β-glycerophosphate and ascorbic acid-2-phosphate, OGM osteogenic media with OPM and 2.5 nmol·L^-1^ rhBMP-6

To assess the cellular basis for the bone formation phenotype, mesenchymal progenitor cells were harvested and evaluated using colony forming unit-fibroblast (CFU-F) and CFU-AP, respectively. Ocn-Cre + Rspo2^f/f^ mice at 1-month of age showed approximately a 50% decrease in both CFU-F and the percent of cells that were CFU-ALP-positive (Fig. 6a, b). Similarly, at 3-months of age Ocn-Cre + Rspo2 f/f mice showed a 33% decrease in progenitor cell colonies, and a 20% decrease in the percentage of ALP-positive progenitor colonies (Supplementary Figures S6).

To assess the functionality of Rspo2-deficient osteoblasts, bone marrow-derived MSC (BM-MSC) were isolated from 1-month-old WT and Ocn-Cre + Rspo2^f/f^ mice. BM-MSC from targeted mice showed decreased mineralization as measured by Alizarin Red S staining (Fig. 6c, d). Similar to cells isolated from Rspo2^Ftl^ mice, there was no difference in ALP staining (data not shown). Cells harvested from mice at 1-month of age showed reductions in Rspo2 at the both the and protein (Fig. 6e) and RNA level (Fig. 6f), although at the gene expression level there were not concomitant changes in Wnt target genes nor osteoblast genes (Fig. 6).

### Ocn-Cre + Rspo2f/f mice show reduced canonical Wnt signaling in bone

**Fig. 7:**
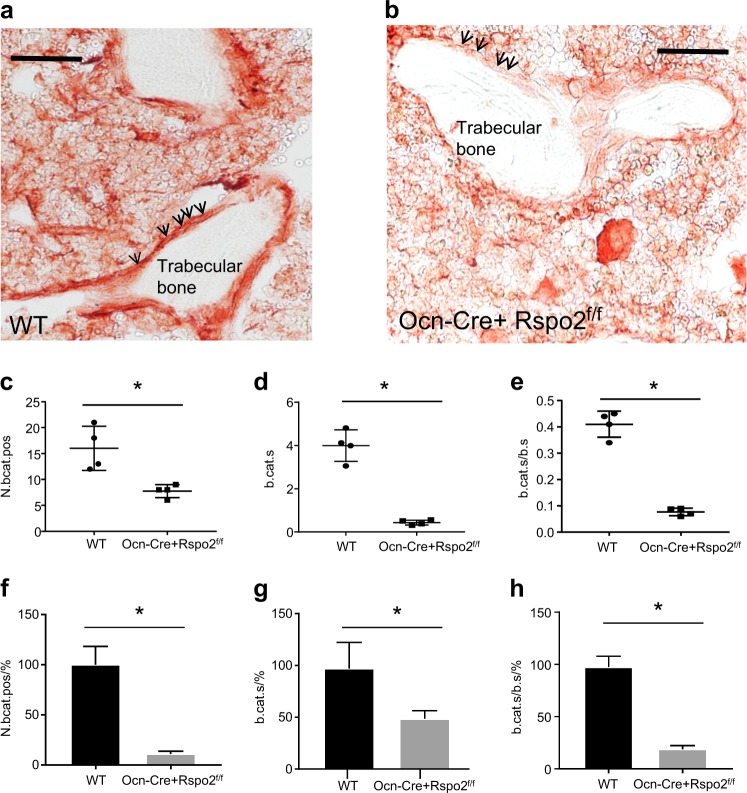
Decreased active-beta catenin staining of bone surfaces from Rspo2-deficient mice. **a**, **b** Representative images of active beta-catenin immunohistochemistry of bones from 3-month-old mice. Arrows indicate bone surfaces and trabecular bone is labeled. Red indicates cell-associated active beta-catenin presence on trabecular bone (**a**), WT, (**b**). Ocn-Cre + Rspo2^f/f^ Scale bar is 100 μmol·L^-1^. **g**, **h** Histomorphometric quantification of beta-catenin positive bone surfaces. **c** Number of active-beta catenin positive cells. **d** Beta-catenin-positive surface. **e** Beta-catenin positive surface relative to total bone surface. **f** Percent Ocn-Cre + Rspo2^f/f^ active-beta catenin positive cells relative to WT. **g** Percent Ocn-Cre + Rspo2^f/f^-positive active-beta catenin surface relative to WT. **h** Percent positive Ocn-Cre + Rspo2^f/f^ active-beta catenin surface per total surface. WT *n* = 3 (2 male, 1 female), Ocn-Cre + Rspo2^f/f^, *n* = 3 (2 male, 1 female). *indicates *P* < 0.05 for genotype groups

Despite the failure to identify statistically significant changes in gene expression from cells in culture, RNA was harvested from the cortical bone of skeletally mature mice 6- to 7-month-old mice. There were statistically significant decreases in Rspo2 (Supplementary Figure S7A) as well as statistically significant decreases in Wnt responsive genes, Axin 2 (Supplementary Figure S7I), Lrp5 (Supplementary Figure S7J), and RNF43 (Supplementary Figure S7K), but not in osteoblast-associated genes (Supplementary Figure S7I-L). Consistent with the reduction in canonical Wnt signaling at the transcriptional level, decreased canonical Wnt signaling was also demonstrated in trabecular bone using immunohistochemistry to assess active beta catenin (Fig. 7). WT mice (Fig. 7a) showed much greater active beta-catenin staining than Rspo2-deficient mice (Fig. 7b). This was enumerated using histomorphometry and there are statistically significant differences in active-beta catenin positive osteoblasts (Fig. 7c, f)), total active-beta catenin surface (Fig. 7d, g), and active-beta catenin surface per total surface (Fig. 7e, h).

## Discussion

Wnt signaling is important in bone development, osteoblastogenesis, and bone maintenance and remodeling, making it a popular target for research on bone anabolic agents. However, differential modulation of the pathway by specific ligands and cofactors, such as R-spondins, is not well-characterized in adult bone. Here we have investigated the role of the Wnt agonist RSPO2 in osteoblastogenesis and in postnatal bone to elucidate its contribution to these processes. We utilized *Rspo2*-null limb bud progenitor cells isolated from Rspo2^ftl^ mice for in vitro investigation. We also developed a novel *Rspo2* conditional knockout mouse model for specific deletion of *Rspo2* using Cre-recombinase.

*Rspo2*-null limb bud progenitor cells have decreased osteoblastogenesis and mineralization, with alterations in an osteogenic gene expression profile that was somewhat surprising. While Osx-expression was low, Runx2 remained unchanged in Rspo2-deficient cells. *Osx*-null mice do not form any bone, yet even the very low *Osx* expression seen in our study is obviously sufficient for some degree of in vitro differentiation.^23^ Downstream osteogenic genes such as *Bsp* show expression levels similar to WT cells. *Ocn* is decreased initially, but its expression reaches levels similar levels to WT cells at later time points in osteoblastogenesis. Relatedly, BM-MSC obtained from Ocn-Cre + Rspo2f/f mice showed reduced osteogenesis, but basal expression of osteoblastic genes was unaltered. While Rspo2 expression is low in cells from recombined mice, there is no change in genes associated with beta-catenin signaling nor with osteoblastogenesis. Notably, RNA from cortical bone does show changes suggestive of alterations in beta-catenin signaling, and there is a reduction in active beta-catenin by immunohistochemistry. Despite the changes in beta-catenin signaling observed in vivo, genes associated with osteoblastogenesis remain unchanged in whole bone, similar to the gene expression analysis of BM-MSC. Future studies will need to more thoroughly dissect the transcriptional program associated with reduced Rspo2. These studies will need to be completed in the presence and absence of Wnt stimulation.

Consistent with some of our gene expression data, *Lrp5* knockout calvarial osteoblasts had no change in the expression of *Runx2*, but did have a significant decrease in the expression of *Osx*.^24^ They also had decreased mineralization with no change in ALP staining, as we observed in the both the *Rspo2*-null limb bud progenitor cells and in BM-MSC. Comparatively, *Lrp6* knockout calvarial osteoblasts had reductions in both mineralization and ALP staining, as well as moderate decreases in the expression of *Runx2*, *Osx*, and *Ocn*, suggesting an even earlier and more sustained role for *Lrp6* in osteoblastogenesis.^24^ Clearly, the Ocn-Cre-driven *Rspo2* knockout shares some characteristics of Lrp5- and Lrp6-deficient mice, which could possibly be due to modulation of both *Lrp5* and *Lrp6* levels at the membrane. In this work we have not attempted to evaluate whether RSPO2 may modulate LRP5 and/or LRP6 specifically, but our data does suggest changes in beta-catenin signaling in vivo.

In vivo, osteocalcin-Cre mediated *Rspo2* knockout resulted in a decrease in overall body mass and femur length. Via μCT analysis, we identified significant reductions in the trabecular bone volume fraction, trabecular thickness, and trabecular number, with modest reductions in cortical bone mass at the 3-month timepoint. The reduced bone mass is most pronounced at earlier timepoints (1 and 3 months of age), corresponding to the periods of highest growth and peak trabecular bone mass in mice.^25^ By 6-months, the differences in trabecular bone have lessened, and the cortical bone is no longer significantly different from WT littermates. Mechanical testing results support the significant differences seen in the cortical bone on microCT at 3 months of age. At 6 months of age, male mice also had significant reductions in the maximum load force and bending stiffness, while the μCT data only showed a trend toward reduction in cortical bone mass. The fact that the mechanical function is significantly different at 6 months, but microCT cortical parameters are not, is intriguing and may reflect that the three-point bending assesses whole bone properties while microCT evaluates a discrete portion of the analyzed cortical bone.

Our histomorphometry data show that Ocn-Cre-driven *Rspo2* deficiency results in decreased mineral apposition and bone formation in 1-month-old mice, despite similar numbers of osteoblasts per bone surface, suggesting a deficiency in osteoblast activity. While there is an equivalent osteoblast/bone surface in the Ocn-Cre + Rspo2^f/f^ mice, because WT mice have so much more bone surface, they do have more total osteoblasts and increases in labeled surfaces. These results correspond to our in vitro mineralization data for both the *Rspo2*-null limb bud progenitor cells (Fig. 1) as well as BM-MSC from the targeted Rspo2^floxed^ mice (Fig. 6). Intriguingly, 3-month-old WT mice no longer show increased MAR, but it is notable that WT mice show a 50% reduction in MAR from 1-month to 3-month, whereas the Ocn-Cre + Rspo2^f/f^ show relatively small changes in MAR from 1-month to 3-months. This likely reflects declining bone formation as WT mice mature. Despite MAR and BFR not being different at 3-months of age, it is noted that there is an increase in serum osteocalcin in WT mice relative to Ocn-Cre + Rspo2^f/f^ mice. This is an interesting observation and may reflect that there is more total bone surfaces with osteoblast activity in the WT than the Ocn-Cre + Rspo2^f/f^. WT mice have a 2.7-fold increase in total mineralizing surface over the Ocn-CreRspo2^f/f^ mice, when mineralizing surface is not considered relative to total bone surface (data not shown).

The Cre mouse used for disrupting Rspo2 in this study was developed by the Clemens laboratory over 15 years ago and has been used extensively to disrupt floxed alleles in studies of the osteoblast lineage.^19^ However, very few Cre mice show perfectly discrete cell-type-specific expression, and indeed studies have shown that Cre expression can occur in cells other than mature osteoblasts. For example, Cre is also expressed in late hypertrophic chondrocytes, which may provide an explanation for the abnormality in the growth plate.^22^ Indeed, deficiency of *Rspo2* in chondrocytes has been reported to decrease chondrocyte proliferation, but differences in protein expression in the hypertrophic zone were not noted.^26^ However, canonical Wnt signaling is known to affect progression of chondrocytes through the growth plate, and, consistent with this, Lrp5 knockout mice also have shortened femoral length.^27^ Given that hypertrophic chondrocytes are known to transdifferentiate to osteoblasts, it is possible that the *Rspo2* deficiency hinders the activation of the pluripotent stem cell programs required for this process.^28,29^ Future studies will need to examine the disruption of Rspo2 using additional promoter systems, and particularly using inducible Cre models, to determine an osteoblast autonomous role for Rspo2.

The overall skeletal phenotype of the Ocn-Cre specific knockout of *Rspo2* is consistent with that seen in other disruptions of canonical Wnt signaling in bone, specifically that of *Lrp5* disruption, albeit somewhat attenuated.^30–32^ Specifically, the Lrp5 knockout mouse has decreased bone mass, decreased mineral apposition rate, decreased osteoblast numbers, and decreased femoral length, all of which are similar to the phenotype seen with osteoblast-specific knockout of *Rspo2*.^27^ Despite these post-natal effects, overall skeletal development, similar to the osteoblast-specific *Rspo2* knockout reported here, but in contrast to the global *Rspo2* knockout, is relatively normal. When the *Lrp5* knockout mouse is crossed with the *Lrp6* knockout mouse to create double knockouts, skeletogenesis defects emerge similar to those seen in the *Cttnb1* (Beta-catenin) and *Rspo2* global knockout, although with different left/right and anterior/posterior patterns.^30,33^ LRP5 and LRP6 are the transmembrane co-receptors mediating canonical Wnt signaling, along with the Frizzled receptors, and are regulated by ZNRF3 and RNF43, which are themselves inhibited by RSPO2.^5,6^ Therefore, the consistency of the Ocn-Cre *Rspo2*-deficient phenotype with Lrp5 deficiency corroborates the hypothesis that RSPO2 modulates canonical Wnt signaling in bone likely through modulation of LRP5 and perhaps LRP6 receptors. While LRP5/6 receptors on osteoblasts were not examined specifically, our data supports decreases in canonical beta-catenin signaling in vivo based on gene expression analysis and immunostaining. Additional studies utilizing combined knockouts of *Rspo2* with these receptors would help elucidate this interaction.

The μCT results show consistently decreased trabecular bone in the 1-, 3-, and 6-month old mice, primarily due to decreased trabecular thickness. In WT bone from 3 to 6 months of age there is a net loss of trabecular bone, while the cortical bone expands.^25^ The phenotype of Rspo2-deficient mice does not increase in severity from 3 to 6 months, perhaps because of the net loss of trabecular bone that is occurring, particularly in WT mice. There is an increase in serum TRAP in 6-month-old mutant mice, but the significance of this is not known. This phenotype may be consistent with the *Wnt10b*-null mouse model in which the growth rate and bone mass are normal at 1 month of age, but progressive osteopenia develops thereafter.^34^ A potential role for Rspo2 in the repression of osteoclastogenesis will need to be investigated further in studies that activate osteoclast activity, such as ovariectomy.

While there is an apparent cortical bone phenotype based on both microCT and mechanical testing, it is arguably less severe than the trabecular phenotype. It is interesting to note that other models of deficient Wnt signaling have shown discrepancies between changes in trabecular and cortical bone. As an example, overexpression of *Wnt10b* increases trabecular bone, with minimal impact on cortical bone.^35^ Further studies to age our novel model to a geriatric timepoint are needed to determine the impact of *Rspo2*-deficieny on both trabecular and cortical bone.

The decreased progenitor cell and pre-osteoblast populations as determined by CFU-F and CFU-AP respectively, are also somewhat unexpected given that osteoblast density is similar. However, as previously mentioned, it is important to realize that despite similar osteoblast density (osteoblasts per bone surface), in both 1-month-old and 3-month-old mice there is greater total osteoblasts. Disruption of canonical Wnt signaling has been shown to decrease progenitor cell number, such as in the *Lrp5*-knockout and the *Wnt10b*-knockout models.^27,34^ However, high levels of expression of stabilized beta-catenin also decreased the number of CFU-F colonies, while lower levels of expression enhanced the proliferation of MSC thereby increasing these numbers.^36^ In another study, addition of WNT3A, a stereotypically canonical Wnt ligand, increased both total number and percentage of ALP-positive colonies, while addition of WNT5A, a stereotypically noncanonical Wnt ligand, had no effect in this assay.^37^ Clearly the tuning of specific Wnt ligands is critical to Wnt signaling’s ultimate effects on progenitor cell renewal, proliferation, and differentiation. In our model, it is possible that a decreased number of osteoblast progenitors contributes to the development of the initial trabecular phenotype. This will require further investigation. Additionally, considering the reduced progenitor cell population, it is conceivable that mice with osteoblast deficiency in *Rspo2*, would have a reduced regenerative capacity when challenged with bone injury or may lose bone more quickly when challenged by aging or ovariectomy.

Together, our data indicate that *Rspo2* modulates osteoblastogenesis and mineralization, both in vitro and in vivo. *Rspo2*-deficiency leads to decreased mineral apposition and bone formation rates, and thus to fewer and smaller trabeculae, decreased overall bone mass, and decreased bone strength. This specific phenotype is similar to other models that perturb the canonical Wnt signaling pathway, particularly those that disrupt receptors LRP5 and LRP6, supporting the canonical Wnt signaling pathway as the primary mechanism of RSPO2 activity in osteoblasts. Indeed, our in vivo results suggest that canonical Wnt signaling through beta-catenin stabilization is reduced in Rspo2-deficient mice. However, the clear differences between our model and previous models that disrupt specific Wnt ligands suggest that RSPO2 modulates the receptors that integrate the signal from many Wnt ligands rather than tuning one specific Wnt ligand-mediated signal. Given our reported reduction in bone mass is more modest compared to some of the other models that disrupt Wnt signaling, this could be beneficial as an option to more carefully tune the activity of osteoblasts therapeutically.^32^ In summary, we have established RSPO2 as an important modulator of bone mass via its regulation of osteoblast function in postnatal bone.

## Materials and methods

### Animals

Animals were group housed in specific-pathogen-free vivaria and all experiments were performed in accordance with institutional policies. Rspo2^ftl^ mice were used for limb bud mesenchymal progenitor cell isolation.^10,11^ Ella-Cre mice were obtained from Jackson Laboratories (Bar Harbor, ME).^38^ Ocn-Cre mice were obtained from Dr. Tom Clemens, Johns Hopkins University.^22^ Mice were humanely euthanized at 1, 3, and 6 months of age for skeletal phenotyping and cell isolation. Mouse harvests and subsequent analyses were done in a blinded, unbiased manner.

### Generation of Rspo2^flox^ mice

To create the *Rspo2* conditional, or floxed, allele (Rspo2^floxed^), we generated a targeting construct in which a 2.9 kb genomic DNA fragment containing exons 4 and 5 was flanked by loxP sites (Fig. 1a). The 5′ homology arm was 2.9 kb in length and the 3′ homology arm was 4.7 kb in length. We used the targeting construct, which also contained a neomycin resistance cassette flanked by Frt sites, to electroporate V6.5 embryonic stem cells (ES cells), which are derived from C57BL/6 × 129/Sv hybrid embryos. ES cell clones were identified as targeted by Southern blot analysis using probes flanking the 5′ and 3′ ends of the targeting construct. ES cell lines were then karyotyped, and one targeted line with no karyotype abnormalities was injected into C57BL/6 embryos to generate chimeric mice. Injection of the successfully targeted ES cells into C57BL/6 blastocysts resulted in 13 chimeric pups, which were crossed to C57BL/6 mice for production of the F1 generation and determination of germline transmission. Of the 43 F1 mice produced, 17 were confirmed via PCR to be heterozygous for the targeted allele for both loxP sites. The construct was determined to be intact via Southern blot analysis for both arms of the targeting construct. The targeting construct was designed with an intronic deletion permitting the use of a single pair of PCR primers flanking the downstream loxP site for genotyping. Subsequently, we crossed Rspo2^flox^ mice with C57BL/6 mice for 4 to 6 generations before crossing them with mice expressing Cre recombinase.

### Cell type-specific deletion of Rspo2^fl^ allele

The Cre-transgenic mice used in this study have been previously described: EIIa-Cre^38^ and Ocn-Cre.^22^ We crossed hemizygous Cre transgenic mice with homozygous Rspo2^flox^ mice to generate heterozygous Rspo2^flox^ offspring with and without a Cre allele. We then crossed homozygous Rspo2^flox^ mice with heterozygous Rspo2^flox^ mice to generate the following offspring: WT mice, mice hemizygous for a Cre allele, mice homozygous for the Rspo2^flox^ allele, hereafter referred to as Rspo2^f/f^, and Rspo2^f/f^ mice that were also hemizygous for a Cre allele. For each age group, mice from multiple litters were utilized. Generally, littermates (raised as cage-mates) were euthanized at indicated ages (1, 3, or 6 months). We genotyped offspring by PCR using the following primer sequences: Cre-for, 5′- TTACATTGGTCCAGCCACC-3′, Cre-rev, 5′-ACCAGCCAGCTATCAACTCG-3′, product size 102 bp; Rspo2-floxB-for, 5′-GCACTGTCCAGGAGGTAGGTCTAAAC-3′, Rspo2-floxB-rev, 5′- CCTTCTTCTGAGCACCATCTGC-3′, product size 359 bp (floxed) and 413 bp (WT). Rspo2-floxB-for and Rspo2-floxB-rev were combined with Rspo2-floxA-for 5′-GACTCTTACTGCCTGGGATCCTCATT-3′ to assess recombination with a product size of 512 bp.

### Skeletal Preparations

Rspo2^f/f^ males were time-mated with Rspo2;^f/+^Ella-Cre + females. e16.5 and e18.5 mouse embryos were harvested, digested, and stained with Alizarin Red S and Alcian Blue by standard methods.^39^

### Micro-computed tomography

Femurs were dissected, cleaned of soft tissue, and wrapped in PBS-soaked gauze. The wrapped femurs were loaded into 9.0 mm diameter scanning tubes and imaged in a µCT scanner (model μCT50, Scanco Medical, Wayne, PA, USA). Femurs were scanned using the following parameters: 6.0 μm isotropic voxel size, 55kVp, 145 μA, 1 000 projections per 180°, and 1 500 ms integration time. Cortical bone parameters were measured by analyzing 50 slices in the mid-diaphysis. This defined region was the central portion between the proximal and distal ends of the femur. A semi-automated contouring method was used to determine the outer cortical bone perimeter, as previously described.^40^ A fixed, global threshold (300 mg HA per cm^3^) of the maximum gray value was used to distinguish bone from soft tissue and marrow. Trabecular bone parameters were measured by analyzing 150 slices of the distal metaphysis, as previously described.^40^ A fixed, global threshold of 220 mg HA per cm^3^ was used to distinguish trabecular bone from soft tissue and marrow. A Gaussian low-pass filter (*σ* = 0.8, support = 1) was used for all analyses. Nomenclature is reported as previously described.^41^

### Histology and Immunohistochemistry

Tibias were dissected and fixed in fresh 4% paraformaldehyde at 4 °C for 24 h and then decalcified in 12% EDTA for 1 week. After decalcification, tibias were embedded in paraffin and 7 µm longitudinal sections were obtained. For staining of tartrate-resistant acid phosphatase (TRAP), after de-paraffinization and rehydration, sections were incubated with acetate buffer containing naphthol-AS-BI-phosphate and Fast Red Violet at 37 °C for 30 min. The sections were then counterstained with 0.1% Fast Green.

For immunohistochemistry, paraffin sections were deparaffinized with Xylenes (Fisher Chemical) and rehydrated and treated with 0.3% Hydrogen Peroxide (SIGMA) in methanol for 30 min to suppress endogenous peroxide activity. Antigen retrieval was achieved by microwaving the sections in 10 mmol·L^-1^ citrate buffer (pH 6.0) for 10 min followed by gradual cooling to room temperature. Sections were incubated overnight at 4 °C with a Non-phospho (Active) β-Catenin (Ser33/37/Thr41) (D13A1) Rabbit mAb #8814, Cell Signaling at 1:200 dilution, and then procedures were followed according to Colorimetric detection UltraVision ONE Detection System HRP Polymer & AEC Chromogen, TL-015-HAJ (Themo Scientific).

### Histomorphometry

All histomorphometry was performed using BIOQUANT OSTEO and all parameters were calculated according to the recommendations of the Histomorphometry Nomenclature Committee of the American Society of Bone and Mineral Research.^42^

For dynamic histomorphometric analysis of bone formation, all animals were labeled with alizarin complexone and calcein. Before euthanasia 1-month-old mice were injected on day 4 and 3-month-old mice were injected on day 8 with alizarin complexone subcutaneously at a dose of 30 mg·kg^-1^. At 1 day before killing, calcein was administered subcutaneously at a dose of 20 mg·kg^-1^. At harvest, right tibiae were stripped of soft tissues, bisected, and immediately fixed in 70% ethanol under vacuum. Tibiae were embedded in methyl methacrylate without decalcification. Sagittal sections of the proximal portion of the tibias were cut at 6 μm thickness, and sections were stained with Goldner’s Trichrome.

TRAP stained images were used to quantify osteoclasts. Briefly, trabecular bone regions of interests (ROI) were selected below the growth plate and contour was drawn along bone surfaces and within bones from wild-type and knockout mice tibias separately total bone surface measured. Specifically, to count osteoclasts, contour was drawn on osteoclasts surface of those bone surfaces and then measured and calculated for osteoclast number, osteoclast surface per bone surface and total bone surface.

Active beta-catenin-positive cells and bone surfaces were counted using images of active beta-catenin Immunohistochemistry. Similar to the procedure used to count osteoclasts, to count b-catenin positive osteoblasts, a contour was drawn along the bone surface and within bone to measure total bone areas and surfaces and then to count active beta-catenin positive straining, those osteoblast bone surfaces were selected and a contour was drawn specifically on osteoblasts showing positive staining and total number of positive osteoblasts, bone surface, and positive surface per bone surface were determined.

### Biomechanical testing

Right femora were subjected to 3-point bending by standard techniques after microCT analysis at the Penn Center for Musculoskeletal Disorders Biomechanics Core. Femora were loaded in the anterior-posterior direction at 0.03 mm·s^-1^ using a electromechanical testing machine (Instron 5542, Instron Inc., Norwood, MA) with the posterior side in tension between lower supports that were 7.66 mm apart for the 1-month samples and 11.79 mm apart for the 3-month and 6-month samples, with the upper loading pin in the center of the lower supports. All bones were tested at room temperature and kept moist with PBS. Crosshead displacement was obtained via the Instron system and load data were collected with a 10 N load cell (Instron Inc., Norwood, MA) at a sampling frequency of 100 Hz. Load-displacement curves were analyzed for whole bone stiffness and ultimate load using custom computational code (MATLAB R2015a; Mathworks Inc., Natick, MA, USA).

### Bone marrow-derived cell culture

Bone marrow cells were harvested from the left femur of 1-, 3-, and 6-month old mice as previously described.^43^ Cells were pelleted and resuspended in mesenchymal stem cell (MSC) media (αMEM supplemented with 10% FBS, L-glutamine, 100 IU·mL^-1^ penicillin, 100 mg·mL^-1^ streptomycin). 4 × 10^6^ cells were plated on 60 mm dishes in duplicate for CFU-F analysis. Colonies were allowed to grow for 12 days and then stained for Alkaline Phosphatase activity with Fast Red as a counterstain. Colonies were counted using a dissecting microscope. The remaining cells were plated on 100 mm dishes for expansion. At the first passage, cells were seeded at 2.5 × 10^4^ cells per cm^2^ into 48-well tissue culture plates and grown to confluence in MSC media. Protein isolation was performed at 24 h for western blotting. At confluence, cells were cultured in osteopermissive media (OPM: αMEM supplemented with 10% fetal calf serum, 100× l-glutamine, 100 IU·mL^-1^ penicillin, 100 mg/mL streptomycin, 32.3 µg·mL^-1^ ascorbic acid 2-phosphate, 5 mmol·L^-1^ β-glycerophosphate). Cells were then fixed and stained with Alizarin red S as previously described.^12^

### Limb bud mesenchymal progenitor cell culture

Heterozygous Rspo2^ftl^ mice were time-mated and embryos were harvested at e16.5. Limbs were dissected from the embryos, stripped of their autopods and soft tissues, and minced. They were then placed in media and aspirated and ejected through an 18-gauge needle 10 times. The media and tissue mixture was then plated on a 10 cm plate and the adherent population was selected. The resultant cells are able to undergo osteogenesis as seen by ALP staining and Alizarin red S staining of mineral, as well as adipogenesis as seen by Oil Red O staining of lipid (data not shown). For osteogenesis, cells were seeded at 2.5 × 10^4^ cells per cm^2^ in 6- or 48-well tissue culture plates. The next day, cells were transferred to osteogenic media (OGM: αMEM supplemented with 10% fetal calf serum, 100× L-glutamine, 100 IU·mL^-1^ penicillin, 100 mg·mL^-1^ streptomycin, 32.3 µg·mL^-1^ ascorbic acid 2-phosphate, 5 mmol·L^-1^ β-glycerophosphate, 2.5 nmol·L^-1^ rhBMP-6 [R&D Systems]). 10 nmol·L^-1^ Recombinant RSPO2 [R&D systems] was added during rescue experiments and refreshed every other day. RNA isolation occurred at 24 h, 5 days, 10 days, and 15 days. ALP staining occurred at 5 and 7 days. Alizarin red S staining of mineral occurred at 10 days, 15 days, and 20 days.

### Western blotting

Cells were lysed in RIPA buffer in the presence of Phosphatase and Protease Inhibitor cocktails (Pierce and Roche, respectively). Lysates were separated on 4%–20% gradient SDS-PAGE gels and transferred to nitrocellulose membranes. After blocking with Odyssey Blocking Buffer (Licor) for 30 min, blots were incubated with the following primary antibodies: Anti-RSPO2 (sc-292494, sc-74883) and Anti-Beta-Tubulin (Sigma T7816). This was followed by incubation with the appropriate secondary antibody (Li-Cor) and imaging on a Li-Cor Odyssey CLx.

### Gene expression

Cells were collected in TRIzol Reagent for RNA isolation and either the Directzol RNA isolation kit (Zymo) or RNeasy kit (Qiagen) were used for RNA purification according to the manufacturer’s instructions. For long bone RNA, tibiae and femora were collected from Rspo2^f/f^ and Ocn-Cre, Rspo2^f/f^ mice from 6–7 month old mice. Bone marrow and soft tissues were removed and clean intact bones were processed. Only the mid diaphyses were used to make RNA. Briefly, to homogenize the bones, the bones were placed in Precellys grinding tubes (Bertin Technologies) in TRIzol Reagent and centrifuged at 6 500 r·min^−1^ 3 × 30 s using Precellys Evolution (Bertin Technologies) according to manufacturer instruction. During the grinding process, cold temperature was maintained in Cryolys filled with dry ice (Bertin Technologies). RNA was then precipitated with ethanol and resuspended in RNase free water, and quantity measured using Nanodrop. Equivalent quantities of RNA were reverse-transcribed to cDNA using the Applied Biosystems High-Capacity cDNA Reverse Transcription kit. Gene expression was quantified using either a ViiA 7 or a 7500Fast Real Time PCR Systems (Applied Biosystems, Foster City, CA, USA) with PowerUp SYBR Green Master Mix or SYBR Select Master Mix (Applied Biosystems). For each gene of interest, samples were analyzed in duplicate or triplicate and control wells were simultaneously analyzed to rule out DNA contamination and primer dimer binding. Proper amplicon production was confirmed by melt curve analysis. Data were normalized to the housekeeping gene 18 S rRNA or beta-actin and presented as fold-change expression relative to WT whole bone, calculated using the formula 2^− ΔΔC(t)^. 18 S and beta-actin C(t) values were stable across treatment groups. Primer sequences are available upon request.

### Measurement of serum markers of bone formation and resorption

Serum was collected by intracardiac puncture at the time of euthanasia and frozen at −70 °C until assayed. Osteocalcin (Mouse osteocalcin-ELISA kit Cat# LS-F5375 LSBio, USA) and TRAP5b (MouseTRAP™ (TRAcP 5b) ELISA Cat# SB-TR103 IDS, immunodiagnosticsystems, USA) in serum of WT and Ocn-Cre + Rspo2^f/f^ 3- and 6-month-old mice were quantified using commercially available kits. For osteocalcin, briefly, a 100 μL of standard or diluted serum samples were added to wells in a 96 well assay plate and incubated for 1 h at 37 °C. After three washes 100 μL of detection Reagent A was added and incubated for 1 h at 37 °C. After washes, detection reagent B was added and incubated at 30 min at 37 °C. After 3 washes, TMB substrate solution was added and incubated at 10–20 min at 37 °C. Stop solution was added and absorbance was read at 450 nm using a spectrophotometer.

For TRAP5b, briefly, A 100 microliters of anti-mouse TRAP antibody was added to wells that were coated with anti-rabbit IgG for 1 h at room temperature. After which, the wells were used and either standard or serum samples (25 μL, undiluted in duplicates) were added to appropriate wells. Releasing reagent was added to treatment wells and following wash steps, a 100 μL of substrate was added to all wells and incubated for 2 h at 37 °C. The reaction was stopped by adding stop solution and absorbance was read at 405 nm in a spectrophotometer.

### Statistics

Two-way ANOVA or Student’s t-test were used to analyze differences in population means, after determining that the data were normally distributed (D’Agostino-Pearson test) and exhibited equivalent variances (F test). All *t* tests were two-sided and unpaired. Tukey corrections were used for multiple comparisons during the two-way ANOVA. *P*-values less than 0.05 were considered statistically significant. While statistically significant differences in bone phenotype exist between male and female mice of both control and Rspo2^f/f^ mice, they are consistent with well-established sex differences in bone geometry in mice and are not reported herein.

### Data availability

All data has been provided within the manuscript and supplementary files.

## Electronic supplementary material

Supplemental Data
