# Supplementary material for: R-spondin-2 is a Wnt agonist that regulates osteoblast activity and bone mass
Source: Bone Res. 2018 Aug 14;6:24. doi: 10.1038/s41413-018-0026-7 (PMC6089978; doi:10.1038/s41413-018-0026-7)
Supplement: Supplementary file 1 — Supplemental Data [file 41413_2018_26_MOESM1_ESM.docx]

**SUPPLEMENTAL DATA for Knight et al.**


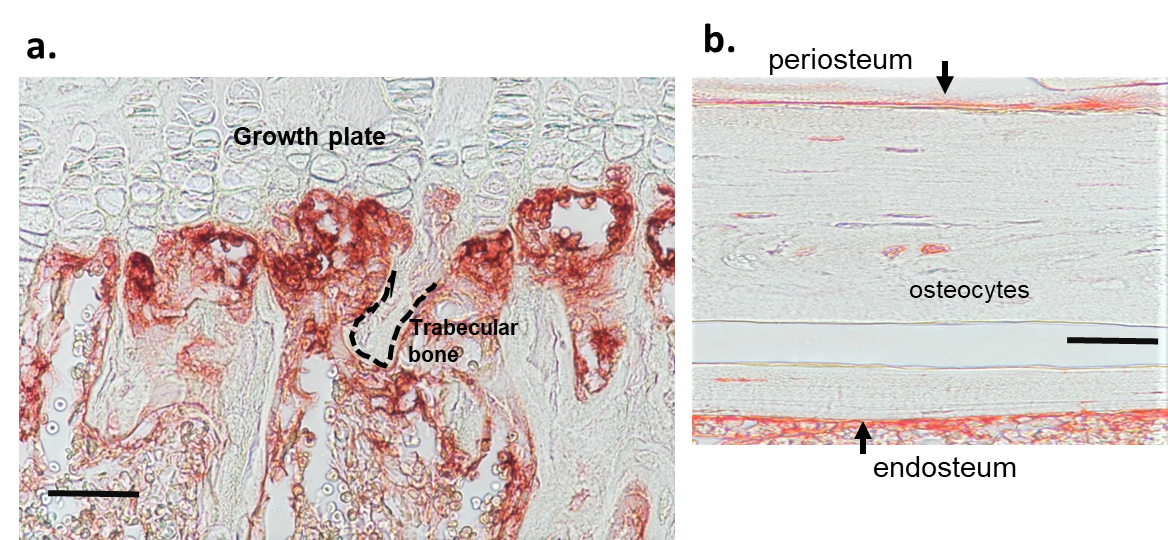


**Supplemental Figure 1. Rspo2 is expressed on bone surfaces and by osteocytes, but is not present in chondrocytes.** Sections were stained with anti-RSPO2 (A) trabecular bone and (B) cortical bone. Red staining indicates positive antibody.

**
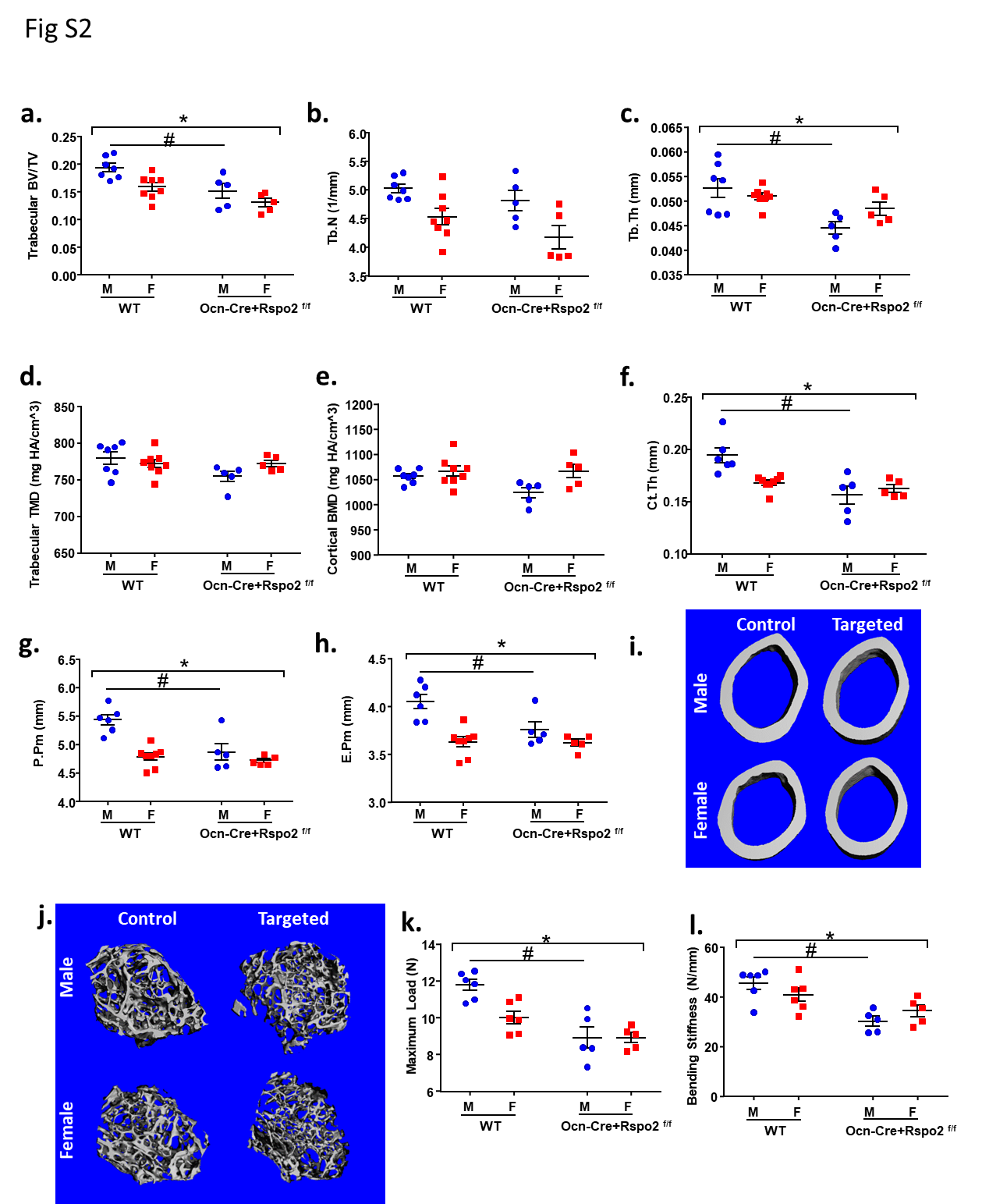
**

**Supplemental Figure 2. Targeted mice at 3-months of age have decreased trabecular and cortical bone parameters.** A-H. Micro-Computed Tomography (uCT) of femurs from 3-month-old mice. Femurs were analyzed for bone volume fraction (A), trabecular number (B), trabecular thickness (C), trabecular bone mineral density (D), cortical bone mineral density (E), cortical thickness (F), periosteal perimeter (G), and endosteal perimeter (H). WT males, n=7; Ocn-Cre+Rspo2^f/f^ males, n=5; WT females, n=8; Ocn-Cre+Rspo2^f/f^ females, n=5. I-J. Three dimensional reconstructions of mid-diaphyseal cortical bone (I) and metaphyseal trabecular bone (J) from representative 3-month old mice. 3-point bending of femurs from 3-month-old mice. Femurs were analyzed for (K) Maximum Load and (L) Bending Stiffness. WT males, n=6; Ocn-Cre+Rspo2^f/f^ males, n=5; WT females, n=6; Ocn-Cre+Rspo2^f/f^ females, n=5. *indicates p<0.05 for genotype groups. # indicates p<0.05 between genotypes within sex.


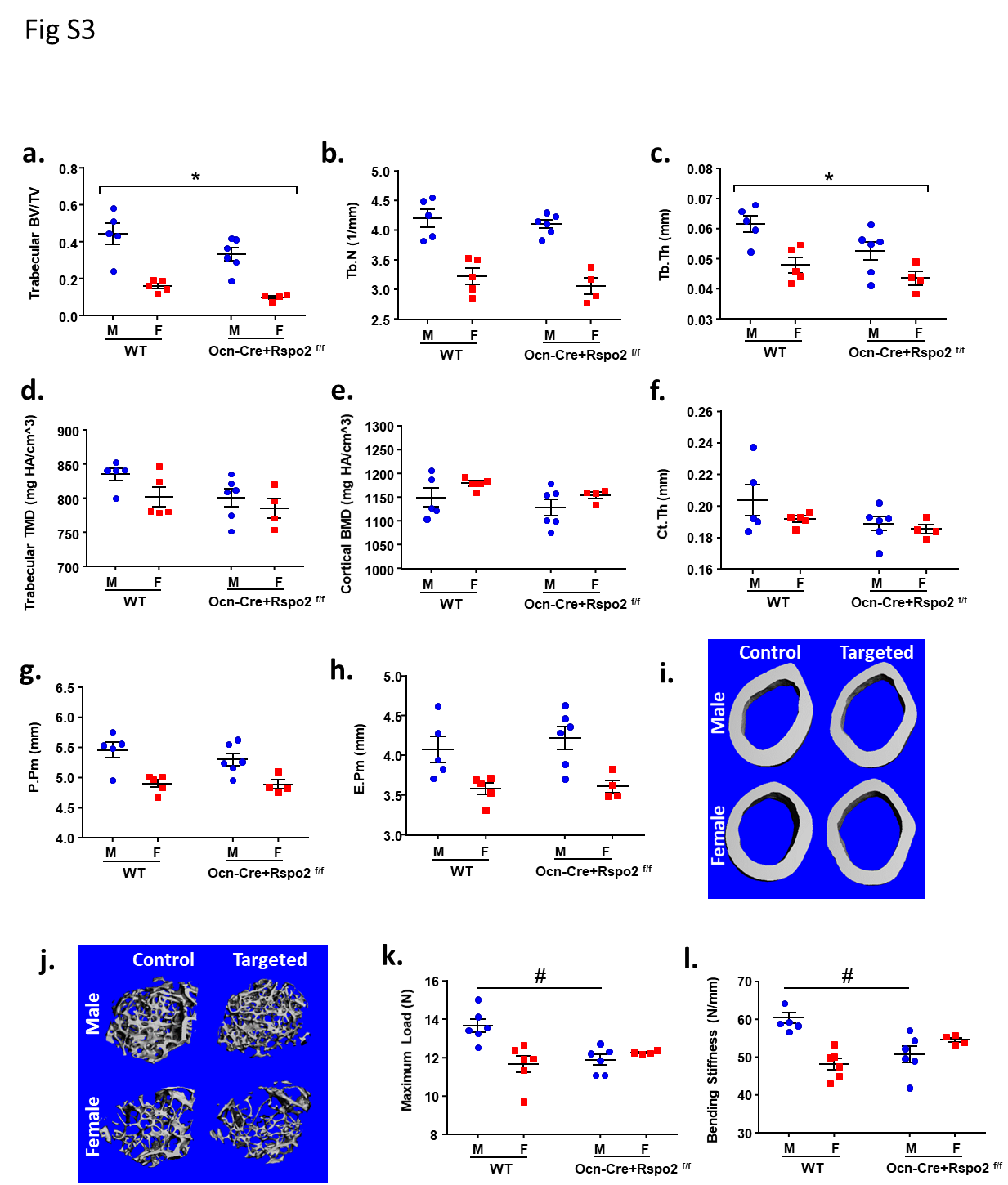


**Supplemental Figure 3. Six-month-old Targeted mice have decreased trabecular and cortical bone parameters.** A-H. Micro-Computed Tomography (uCT) of femurs from 6-month-old mice. Femurs were analyzed for bone volume fraction (A), trabecular number (B), trabecular thickness (C), trabecular bone mineral density (D), cortical bone mineral density (E), cortical thickness (F), periosteal perimeter (G), and endosteal perimeter (H). WT males, n=5; Ocn-Cre+Rspo2^f/f^ males, n=6; WT females, n=5; Ocn-Cre+Rspo2^f/f^ females, n=4. I-J. Three dimensional reconstructions of mid-diaphyseal cortical bone (I) and metaphyseal trabecular bone (J) from representative 6-month old mice. 3-point bending of femurs from 6-month-old mice. Femurs were analyzed for (K) Maximum Load and (L) Bending Stiffness. WT males, n=6; Ocn-Cre+Rspo2^f/f^ males, n=6; WT females, n=6; Ocn-Cre+Rspo2^f/f^ females, n=4. *indicates p<0.05 for genotype groups. # indicates p<0.05 between genotypes within sex.


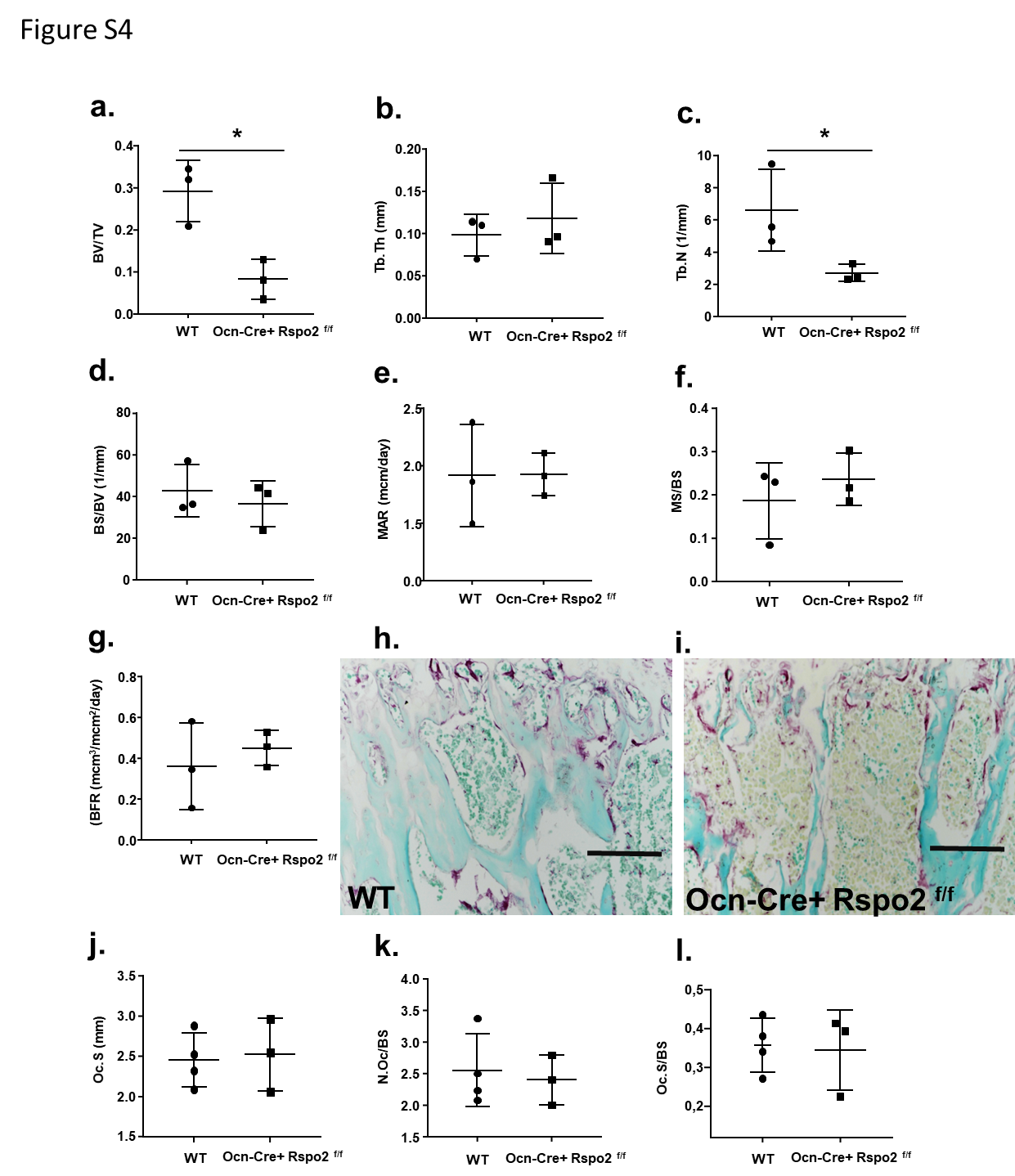


**Supplemental Figure 4. Three-month-old mice do not show increases in mineral apposition (MAR) and bone formation rate (BFR), nor are there changes in osteoclast surface.** Histomorphometric parameters from 3-month-old mice. Tibiae were analyzed for bone volume fraction (A), trabecular thickness (B), trabecular number (C), bone surface (D), mineral apposition rate (E), mineralizing surface (F), bone formation rate (G). WT, n=3 (male); Ocn-Cre+Rspo2^f/f^ n=3 (1 male; 2 female) *indicates p<0.05 for genotype groups. H-I Representative TRAP staining of WT (H) and Rspo2-null slides (I). Pink staining indicates osteoclasts. J-L. Histomorphometric parameters, Osteoclast surface (J), Number of osteoclasts per bone surface (K), Osteoclast surface relative to total bone surface (L) WT, n=4 (3 male; 1 female); Ocn-Cre+Rspo2^f/f^ n=4 (2 male; 2 female).

**
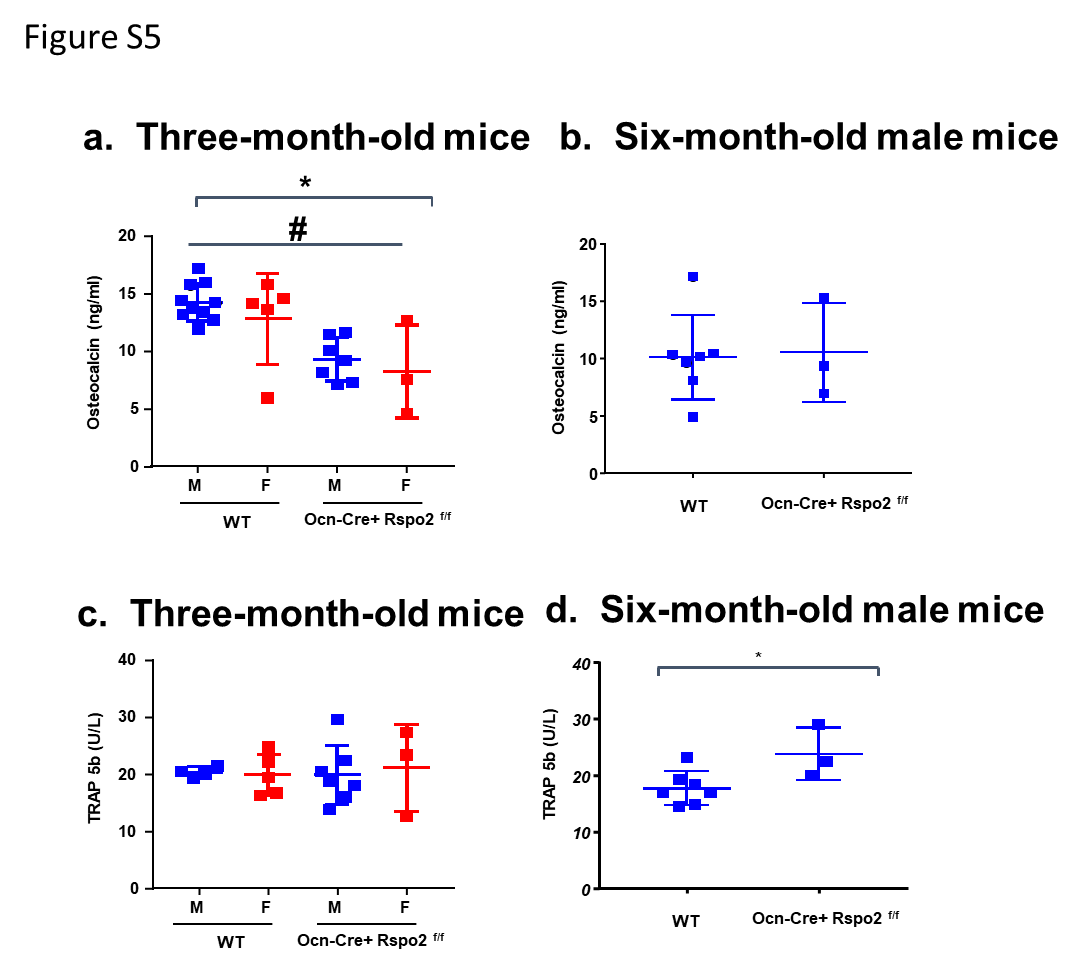
**

**Supplemental Figure 5. Alterations in serum parameters of bone formation and resorption.** Serum was harvested and analyzed by ELISA for Osteocalcin (A,B) and TRAPb (C,D). Males = blue circles, Females = red squares. (A) Osteocalcin in 3-month-old mice (B) Osteocalcin in 6-month-old male mice. (C) TRAPb in 3-month-old mice. (D) TRAPb in 6-month-old mice. For 3-month-old animals (A,C), WT males, n=10; Ocn-Cre+Rspo2^f/f^ males, n=7; WT females, n=5; Ocn-Cre+Rspo2^f/f^ females, n=3. For 6-month-old animals (B,D) only males were analyzed, WT males, n=7; Ocn-Cre+Rspo2^f/f^ males, n=3 *indicates p<0.05 for genotype groups. # indicates p<0.05 between genotypes within sex.

**
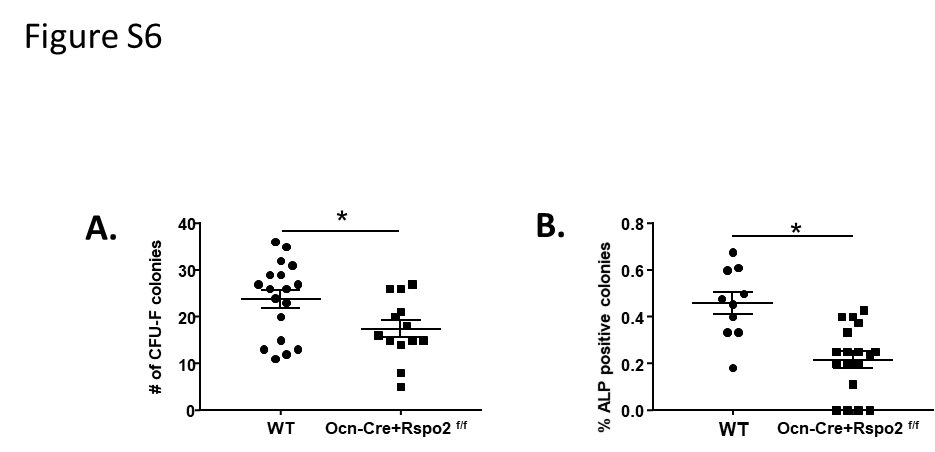
**

**Supplemental Figure 6.** **Reduced CFU-F in three-month old** **Ocn-Cre+Rspo2^f/f^ mice.** A. Quantification of total number of colonies from bone marrow flushed from 3-month-old mice. B. Quantification of percent ALP-positive colonies from bone marrow flushed from 3-month-old mice. WT, n=18 (n=9 males; n=9 females); Ocn-Cre+Rspo2^f/f^, n=13 (n=7 males; n=6 females). *indicates p<0.05.

**
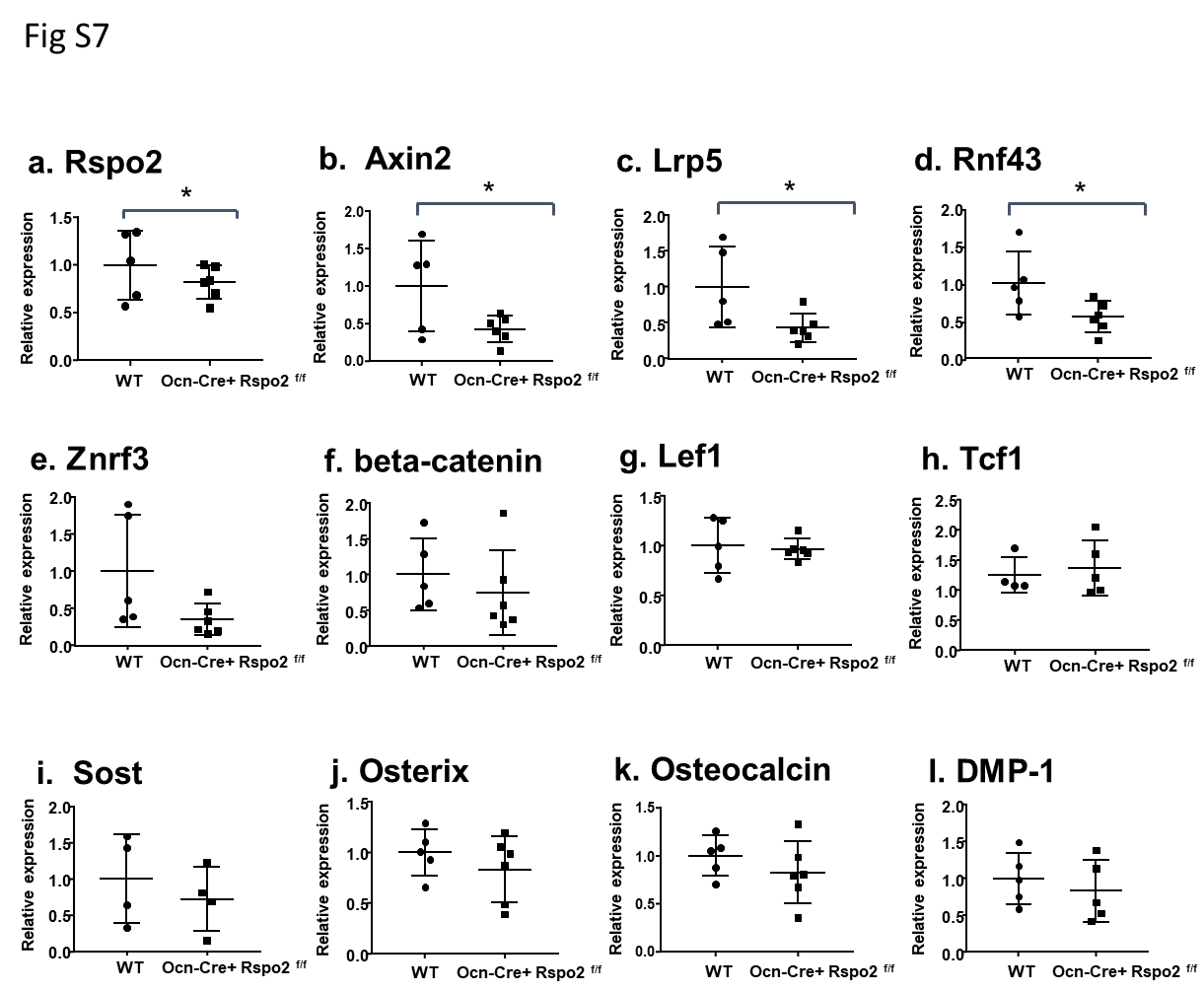
**

**Supplemental Figure 7. Ocn-Cre+Rspo2^f/f^ mice show reduced Rspo2 and beta-catenin signaling in bone.** (A-L) Quantification of expression of Rspo2 (A), genes modulated by canonical Wnt signaling (B-I), and genes involved in osteoblast activity (I-L) from n=5 WT (n=1 male and n=4 female) and n=6 Ocn-Cre+Rspo2^f/f^ (n=4 male and n=2 female) mice at 6-7 months of age. A. Rspo2 expression. B. Axin2 expression. C. Lrp5 expression. D. Rnf43 expression. E. Znf3 expression. F. beta-catenin expression. G. Lef1 expression. H. Tcf1 expression. I. Sost expression. J. Osterix expression. K. Osteocalcin expression. L. Dmp1 expression. *indicates p<0.05.
